# Semisynthesis of A6–A11 lactam insulin

**DOI:** 10.1002/psc.3542

**Published:** 2023-09-11

**Authors:** Rong Xu, Edwina Jap, Ben Gubbins, Christoph E. Hagemeyer, John A. Karas

**Affiliations:** ^1^ Australian Centre for Blood Diseases Monash University Melbourne Victoria 3004 Australia; ^2^ School of Chemistry The University of Melbourne Melbourne Victoria 3010 Australia

**Keywords:** cystine mimetic, diabetes, disulfide bond, insulin, peptide synthesis, semisynthesis

## Abstract

Insulin replacement therapy is essential for the management of diabetes. However, despite the relative success of this therapeutic strategy, there is still a need to improve glycaemic control and the overall quality of life of patients. This need has driven research into orally available, glucose‐responsive and rapid‐acting insulins. A key consideration during analogue development is formulation stability, which can be improved via the replacement of insulin's A6–A11 disulfide bond with stable mimetics. Unfortunately, analogues such as these require extensive chemical synthesis to incorporate the nonnative cross‐links, which is not a scalable synthetic approach. To address this issue, we demonstrate proof of principle for the semisynthesis of insulin analogues bearing nonnative A6–A11 cystine isosteres. The key feature of our synthetic strategy involves the use of several biosynthetically derived peptide precursors which can be produced at scale cost‐effectively and a small, chemically synthesised A6–A11 macrocyclic lactam fragment. Although the assembled A6–A11 lactam insulin possesses poor biological activity in vitro, our synthetic strategy can be applied to other disulfide mimetics that have been shown to improve thermal stability without significantly affecting activity and structure. Moreover, we envisage that this new semisynthetic approach will underpin a new generation of hyperstable proteomimetics.

## Graphical abstract

Proof of principle is provided for the semisynthesis of stable insulin analogues bearing nonnative A6–A11 cystine isosteres. Several biosynthetically derived peptide precursors and a small, chemically synthesised A6–A11 macrocyclic lactam fragment are used. This new semisynthetic approach will support a new generation of hyper‐stable proteomimetics.

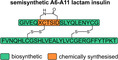

## 1 INTRODUCTION

Insulin replacement therapy is essential for the effective management of type 1 diabetes. Patients with type 2 diabetes also require insulin if they develop β‐cell failure. But despite the relative success of subcutaneously administered basal/bolus insulin dosage regimens in controlling glycaemic levels, significant challenges remain to optimise the effective treatment of diabetes worldwide, such as improving glycaemic control, increasing therapeutic compliance, mitigating weight gain and enhancing the overall quality of life for patients.^1^ To address these issues, orally available,^2^ glucose‐responsive,^3^ rapid‐acting^4^ and ultralong‐acting^5^, ^6^ insulin analogues are currently under investigation. A key factor to consider when developing new insulin analogues is formulation stability, as chemical degradation (e.g., disulfide shuffling, deamidation at A21 and/or B3) and physical degradation (aggregation) can occur.^7^, ^8^, ^9^, ^10^ The stability issue can be particularly problematic for rapid‐acting analogues that are used in insulin pumps and is also important for orally available nanoparticle‐based formulations that are currently undergoing preclinical evaluation.^11^ Moreover, subcutaneously administered insulin analogues with improved physico‐chemical stability would be ideal for developing countries that have only a limited cold chain distribution network.^12^

**FIGURE 1:**
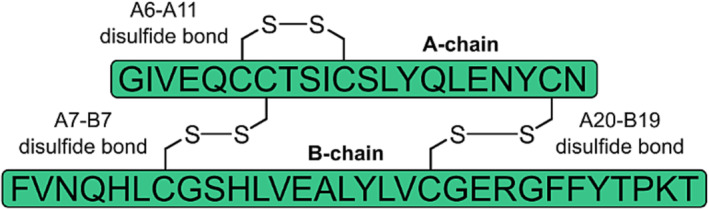
Structure of native human insulin, with its two peptide chains and three disulfide bonds.

Insulin comprises two polypeptide chains, an A‐ and a B‐chain, that are linked by two intermolecular disulfide bonds, with a third intramolecular disulfide bond in the A‐chain (Figure 1). These covalent crosslinks are essential for maintaining its tertiary structure and hence biological activity. Several strategies have been adopted to improve insulin stability and suppress aggregation, such as glycosylation^13^ and the development of single‐chain proinsulin‐like analogues.^14^ Substitution of insulin's A6–A11 disulfide bond with stabilising isosteres such as diselenide,^15^ thioether,^16^, ^17^ methylene thioacetal^18^ and dicarba^19^ bridges has also been investigated; these nonnative cystine mimetics are less susceptible to reduction, disulfide shuffling and subsequent oligomerisation. Furthermore, these modifications do not introduce additional steric bulk to the insulin scaffold and generally impart only minimal structural perturbations, resulting in almost native‐like binding to the insulin receptor (IR). These stabilised, highly potent analogues would therefore be ideal as rapid‐acting therapeutics such as those used in insulin pumps.

Unfortunately, existing methods for their preparation involve extensive chemical synthesis due to the presence of the nonnative crosslinks; therefore, only milligram quantities can currently be isolated.^20^ Even semisynthesis via the incorporation of recombinant B‐chain still requires total chemical synthesis of the A‐chain, which involves well over 40 chemical transformations. This scalability issue significantly hampers their therapeutic potential as recombinant protein synthesis is the only proven method for producing insulin therapeutics on a large scale.^21^ Therefore, to improve the viability of these unnaturally modified insulin analogues as drug leads, new semisynthetic methods with minimal chemical steps are required for their efficient preparation at scale.

**SCHEME 1:**
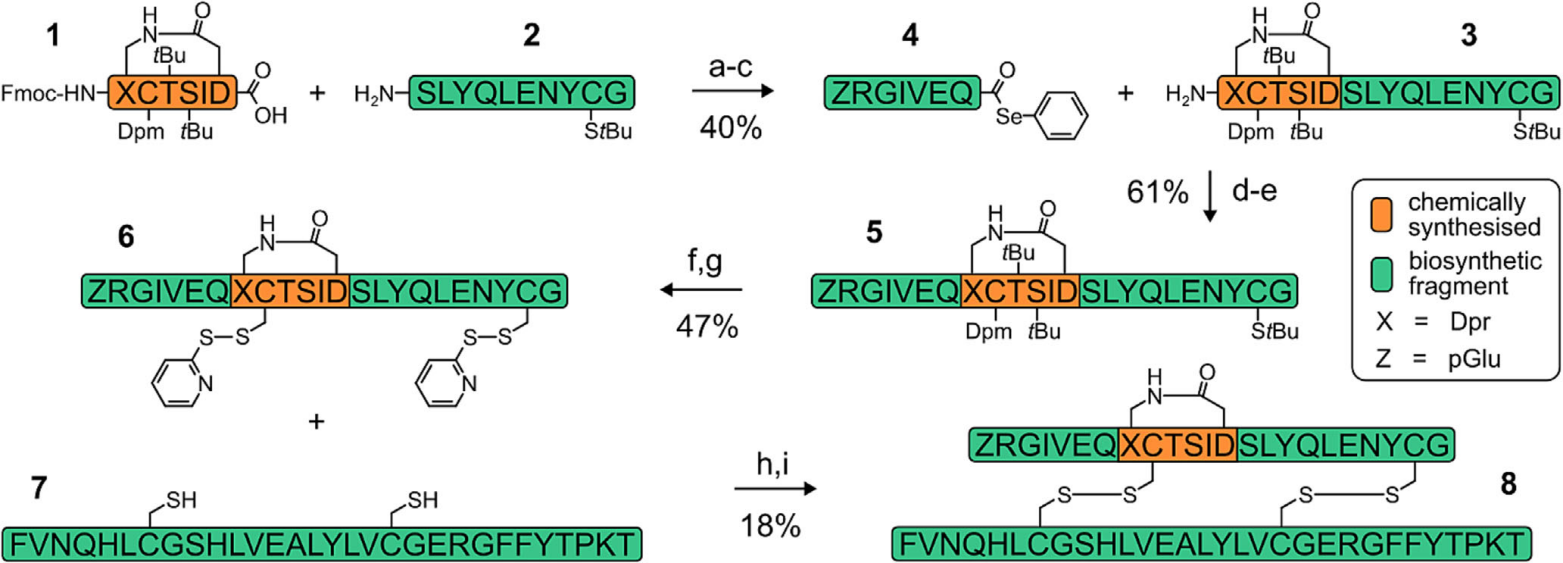
Synthesis of the A6–A11 lactam insulin. (A) 1 eq. COMU, 10 equation *N*‐methylmorpholine, DMF, 25°C, 18 h (**1** pre‐activated). (B) 30 eq. piperidine, 10 min. (C) Preparative RP‐HPLC. (D) 10 equation *N*‐methylmorpholine, DMF, 25°C, 2 h. (E) Preparative RP‐HPLC. (F) 10 eq. DPDS, 2% H_2_O, 2% thioanisole, 96% TFA, 25°C, 1 h. (G) Preparative RP‐HPLC. (H) Aqueous 6 M guanidinium hydrochloride, 0.2 M Na_2_HPO_4_, pH 7, 25°C, 30 min. (I) Preparative RP‐HPLC.

Herein, we demonstrate proof of principle for assembling an insulin analogue bearing a stable crosslink in place of the A6–A11 disulfide bond, via semisynthesis. For this study, a lactam bridge was introduced, which can be readily incorporated using commercially available precursors. To mimic cystine, 4‐atom lactam bridges can be incorporated through the use of aspartyl/diaminopropionyl (Dpr) pairings.^22^ Intermolecular lactam bridges have previously been incorporated into the related insulin‐like peptide 3 (INSL3), whereby moderate to good binding affinity for the INSL3 receptor is still maintained.^23^ We show that A6–A11 lactam insulin can be prepared efficiently from several peptides (A1–A5, A12–A21 and B1–B30) that can be derived biosynthetically and a chemically synthesised A6–A11 fragment bearing the nonnative crosslink (Scheme 1).

## 2 MATERIALS AND METHODS

### 2.1 Materials

Synthesis of peptide precursors was performed manually using fritted syringes on a vacuum manifold via 9‐fluorenylmethyloxycarbonyl (Fmoc) solid‐phase peptide synthesis (SPPS). All reagents were purchased from commercial sources and used without further purification. 2‐Chlorotrityl chloride resin (sub. = 1.2 mmol/g), Wang resin (sub. = 0.73 mmol/g), Oxyma Pure and all standard Fmoc amino acids were sourced from Mimotopes Pty Ltd (Australia). Fmoc‐L‐Dap(Boc)‐OH and Fmoc‐L‐Asp‐OMe were obtained from AK Scientific, Inc. (USA). Fmoc‐Cys(S*t*Bu)‐OH was sourced from CreoSalus, Inc. (USA). (1‐Cyano‐2‐ethoxy‐2‐oxoethylidenaminooxy)dimethylamino‐morpholino‐carbenium hexafluorophosphate (COMU), 1‐[bis (dimethylamino)methylene]‐1*H*‐1,2,3‐triazolo[4,5‐*b*]pyridinium‐3‐oxide hexafluorophosphate (HATU) and 2,2′‐dipyridyldisulfide (DPDS) were purchased from Combi Blocks (USA). Diisopropylcarbodiimide (DIC), 4‐dimethylaminopyridine (DMAP), diphenyldiselenide, acetoacetone, triisopropylsilane (TIPS), thioanisole, 2,2′‐(ethylenedioxy)diethanethiol (DODT), trifluoroacetic acid (TFA), piperidine, diisopropylethylamine (DIEA), *N*‐methylmorpholine (NMM), hydrazine hydrate, *tris*‐carboxyethylphosphine hydrochloride (TCEP.HCl), iodine, dimethylformamide (DMF), dichloromethane (DCM), diethyl ether, acetonitrile and trypsin were purchased from Sigma‐Merck (Australia).

### 2.2 Peptide synthesis

The A12–A21 fragment was assembled on a CEM Liberty Blue microwave peptide synthesiser (USA) using Oxyma Pure/DIC coupling conditions at 90°C. The ZR‐A1‐A5 selenoester and A6–A11 lactam analogues were assembled manually on a Preppy 12‐port manifold in fritted syringes. All yield calculations factored in the mass attributable to TFA counterions.

### 2.3 Analytical RP‐LCMS

All peptides were analysed on an Agilent 1260 Infinity II LCMS (Germany), using an Agilent Pursuit XRs 3 C18 50 × 2.0 mm column, except for the chain combination steps, whereby a Phenomenex bioZen 3.6 μm Intact XB‐C8 50 × 2.1 mm column was used. The mobile phases were as follows: buffer A = 0.05% TFA in H_2_O and buffer B = 0.05% TFA in acetonitrile. Flow rate = 0.4 mL/min, wavelength = 215 nm. The gradient used was 5%–95% buffer B over 9 min. High‐resolution mass spectrometry was conducted on a ThermoFisher Scientific OrbiTrap Exactive instrument (USA).

### 2.4 Preparative RP‐HPLC

All peptides were purified on an Agilent 1260 Infinity II LC system (Germany) using a Phenomenex Gemini 5 μm C18 110 Å 150 × 21.2 mm column. The mobile phases were as follows (unless otherwise specified): buffer A = 0.05% TFA in H_2_O and buffer B = 0.05% TFA in acetonitrile. Flow rate = 10 mL/min, wavelength = 225 nm. All clean fractions were pooled and lyophilised on a Labconco Free Zone (4.5 L, −105°C) freeze dryer (USA). Various linear gradients were used to isolate the desired peptide.

### 2.5 Circular dichroism (CD) spectroscopy

All A6‐A11 lactam insulin isomers and recombinant native human insulin were analysed on an Applied Photophysics Chirascan CD spectrometer. Each sample was dissolved in milli‐Q water and dissolved to a concentration of approximately 0.2 mg/mL.

### 2.6 Insulin Receptor (IR) and Akt phosphorylation assays

Activation of the p‐IR and p‐Akt was assessed by western blotting as previously described.^24^ In brief, HepG2 cells were seeded in six well plates at 0.5 × 10^6^/well 48 h prior to experiments. About 24 h prior to the treatment, culture medium was replaced with serum‐free Dulbecco's Modified Eagle Medium (DMEM) for starvation. HepG2 cells were stimulated with 100 nM insulin and insulin analogues for 15 min, respectively. Cells were washed with phosphate buffered saline (PBS), before being lysed with RIPA buffer containing protease and phosphatase inhibitors (Roche, #04693132001 and #04906837001). After 30 min of centrifugation at 16,000*g*, 4°C, the supernatant was collected and total protein concentration was determined using the bicinchoninic acid (BCA) assay (Thermo Scientific), before the supernatant was added to 6 × sodium dodecyl sulfate (SDS) sample buffer with dithiothreitol (DTT) and heated at 95°C for 10 min. Cell lysates (60 μg total protein) were subjected to SDS polyacrylamide gel electrophoresis (SDS‐PAGE) and transferred to a polyvinylidene fluoride (PVDF) membrane (Millipore). The membrane was blocked with 5% skim milk in Tris‐buffered saline  containing 0.05% Tween 20 (TBST). The membrane was then probed with rabbit Phospho‐IR (Tyr1361) (cell signalling, #3023) or rabbit IR (pan) (cell signalling, #2341) or rabbit Phospho‐Akt (Ser473) (cell signalling, #4060) or rabbit Akt (pan) (cell signalling, #4691) (both diluted 1:1000). Membrane was washed three times with TBST before incubation with 1:5000 goat anti‐rabbit horseradish peroxidase (HRP) secondary antibody (Invitrogen, #65‐6120). Membrane was washed three times with TBST. Chemiluminescence was developed (Thermo Scientific) and imaged (ChemiDoc, Bio‐Rad). The quantification of blots was achieved using Image Lab 6.1 (Bio‐Rad).

### 2.7 Thermal stability assay

All peptides were dissolved in PBS solution (pH 7.4), followed by incubation at 70°C in a water bath. Aliquots were sampled in triplicate at the following time points: 0, 6, and 12 h. Each sample was analysed via LCMS, and the area under the curve of the extracted ion chromatogram of the dominant [M+6H^+^]^6+^ was determined. The data were processed with GraphPad Prism; the error bars indicate one standard deviation from the mean.

### 2.8 Synthesis of protected A6 × A11 lactam (**1**)

**SCHEME 2:**
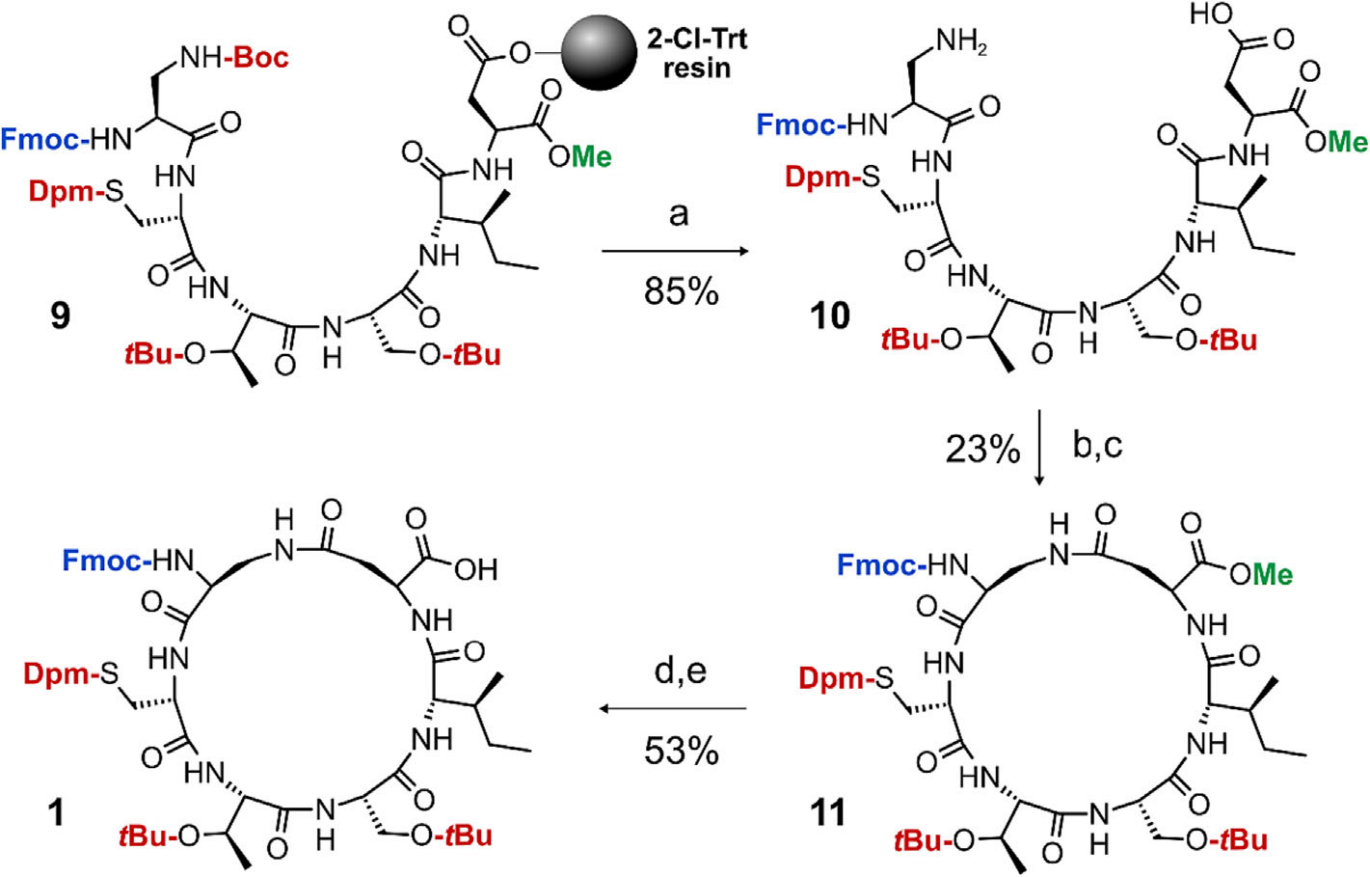
Synthesis of the protected A6–A11 lactam fragment. (A) 3% TFA, CH_2_Cl_2_, 25°C, 18 h. (B) 2 eq. COMU, 5 eq. DIEA, CH_2_Cl_2_, 25°C, 2 h. (C) Preparative RP‐HPLC. (D) 5 eq. SnMe_3_OH, dichloroethane, 55°C, 24 h. (E) Preparative RP‐HPLC.

The peptide was prepared as detailed in Scheme 2. Linear peptide (**9**) was assembled manually on a 0.3 mmol scale using 2‐chlorotrityl resin as the solid support. Fmoc‐L‐Asp‐OMe was first anchored through its sidechain, followed by SPPS using 1 mmol of Fmoc amino acid, 1 mmol of Oxyma Pure and 1.5 mmol DIC in DMF at 50°C. Fmoc deprotection was achieved using 20% piperidine in DMF for 10 min at 50°C. After solid‐phase assembly, the resin was washed with 3% TFA in CH_2_Cl_2_ (10 × 10 mL) into a round bottomed flask, then stirred for 18 h. The solvent was then reduced in vacuo, followed by dissolution in 50:50 acetonitrile/H_2_O and lyophilisation. The crude mass of **10** that was recovered was 320 mg, suggesting a yield of 85% based on the synthetic scale. [M+H^+^]^+^_(th)_ = 1139.4, [M+H^+^]^+^_(exp)_ = 1138.4.

Crude peptide **10** (320 mg, 0.256 mmol) was then dissolved in 95% CH_2_Cl_2_ containing 5% DMF (160 mL). DIEA (445 μL, 2.56 mmol) was then added, followed by the addition of HATU (291 mg, 0.768 mmol); the reaction mixture was stirred for 18 h. The solvent was then reduced in vacuo and the remaining DMF aspirated with nitrogen gas in a warm water bath. The solid was then dissolved in 50:50 acetonitrile/H_2_O (25 mL), then purified in three batches (55%–95% buffer B over 40 min). The clean fractions were pooled and lyophilised. About 68 mg of a white solid was obtained (**11**), indicating a yield from the crude starting material of 23%. [M+H^+^]^+^_(th)_ = 1121.4, [M+H^+^]^+^_(exp)_ = 1120.4.

Trimethyl tin hydroxide (48 mg, 0.2635 mmol) was dissolved in dichloroethane (10 mL), followed by the addition of protected peptide **11** (59 mg, 0.0527 mmol). The reaction mixture was stirred for 24 h at 55°C, followed by washing with 5 mM HCl_(aq)_ (1 × 30 mL) and then aspiration with nitrogen gas to remove the solvent. The residue was then redissolved in 60% acetonitrile_(aq)_, filtered then injected onto the HPLC column, using a gradient of 45%–95%B over 50 min. The fractions were pooled then lyophilised to afford 31 mg of the free acid protected peptide **1** (yield = 53%). The total yield of the A6–A11 fragment as calculated from the crude linear material was 12%. [M+H^+^]^+^_(th)_ = 1107.4, [M+H^+^]^+^_(exp)_ = 1106.4.

### 2.9 Synthesis of S*t*Bu protected A12–A21 (**2**)

The peptide was assembled via microwave assisted SPPS on Wang resin preloaded with Fmoc‐Gly‐OH; Fmoc‐L‐Cys (StBu)‐OH was incorporated at position A20. The peptide was cleaved from the resin with a cocktail of TIPS/H_2_O/TFA (2.5%/2.5%/95%) followed by filtration, nitrogen gas aspiration to reduce the volume, precipitation with diethyl ether, centrifugation, then decanting, to afford 332 mg of the crude peptide (yield = 92.5%). The peptide was purified in two batches using the following buffer system: buffer A = 10 mM NH_4_OAc at pH 8.5 and buffer B = 10 mM NH_4_OAc in 80% acetonitrile_(aq)_ at pH 8.5. The gradient was 20%–80% buffer B over 60 min. About 84 mg of purified peptide **2** was recovered, indicating a yield of 24% based on the synthesis scale. [M+H^+^]^+^_(th)_ = 1278.5, [M+H^+^]^+^_(exp)_ = 1277.6.

### 2.10 Synthesis of protected A6–A21 lactam (**3**)

The A6–A11 lactam peptide **1** (14.0 mg, 12.7 μmol) was dissolved in dry DMF, followed by the addition of COMU (5.4 mg, 12.7 μmol) and then NMM (7.0 μL, 63.5 μmol). The solution was shaken for 3 min, followed by the addition of the A12–A21 fragment **2** (17.6 mg, 12.7 μmol) which was poorly soluble in DMF. The total DMF volume was 2.5 mL. The reaction was shaken for 18 h and monitored by LCMS. Piperidine was then added, and the solution shaken for a further 10 min. The tube was then immersed in warm water and aspirated with nitrogen gas for 1 h to remove the DMF. The oily residue was then dissolved in acetonitrile/H_2_O (50:50) and purified via HPLC. Conditions: 40%–90% buffer B over 50 min. About 11.4 mg of pure A6–A21 protected lactam peptide **3** was obtained, indicating a yield of 40%. [M+2H^+^]^2+^_(th)_ = 1072.8, [M+2H^+^]^2+^_(exp)_ = 1072.4.

### 2.11 Synthesis of ZR‐A1–A5 selenoester (**4**)

The peptide was synthesised on a 0.5 mmol scale using Oxyma/DIC couplings. The hydrazine resin was prepared first on 2‐chlorotrityl resin (using 5 eq. of hydrazine hydrate, 5 eq. of DIEA in DMF), followed by assembly, then cleavage with TIPS/H_2_O/TFA (10 mL). About 375 mg of crude peptide was isolated, which was subsequently purified in three batches (5%–65% buffer B over 60 min). After pooling of the clean fractions and lyophilisation, 102 mg of the ZR‐A1‐A5 hydrazide peptide was obtained, indicating a yield of 19% based on the synthesis scale. The purified hydrazide peptide (50.0 mg, 47.4 μmol) was then dissolved in 0.2 M HEPES, 6 M guanidine hydrochloride, pH 2 (4.5 mL), followed by the addition acetonitrile (500 μL). Diphenyldiselenide (44.4 mg, 142 μmol) and TCEP.HCl (118.6 mg, 474 μmol) were then added, followed by acetoacetone (48.7 μL, 474 μmol). The solution was shaken for 2 h, then filtered and injected onto the RP‐HPLC column for purification (10%–70% buffer B over 60 min). About 40.2 mg of selenoester peptide **4** was obtained, indicating a yield of 80% as calculated from the purified hydrazide peptide. [M+H^+^]^+^_(th)_ = 951.0, [M+H^+^]^+^_(exp)_ = 952.4.

### 2.12 Synthesis of protected ZR‐A1–A21 lactam insulin (**5**)

A6–A21 protected lactam peptide **3** (10.5 mg, 4.65 μmol) was dissolved in dry DMF, followed by the addition of NMM (5.1 μL, 46.5 μmol). The ZR‐A1‐A5 selenoester peptide **4** was then introduced (8.9 mg, 8.37 μmol), and the solution was shaken for 4 h, followed by aspiration with nitrogen gas for 1 h to remove the DMF. The oily residue was then solubilised in acetonitrile/H_2_O, filtered and injected onto the RP‐HPLC column for purification (30%–90% buffer B over 60 min). About 8.7 mg of the protected intermediate peptide **5** was recovered, indicating a yield of 61%. [M+2H^+^]^2+^_(th)_ = 1469.7, [M+2H^+^]^2+^_(exp)_ = 1469.2.

### 2.13 Synthesis of *bis*‐SPy ZR‐A1–A21 lactam (**6**)

Protected ZR‐A1–A21 **5** (5.0 mg, 1.65 μmol) was then treated with a cleavage cocktail of 1% H_2_O and 1% thioanisole in TFA (1 mL), which contained DPDS (3.6 mg, 16.5 μmol). The reaction mixture was shaken for 30 min, followed by the addition of diethyl ether to precipitate the peptide and then centrifugation. The supernatant was decanted, and the white pellet was solubilised and injected directly onto the HPLC column for purification (20%–80% buffer B over 60 min). About 2.3 mg of *bis*‐SPy ZR‐A1 A21 lactam **6** was obtained, indicating a yield of 47%. [M+2H^+^]^2+^_(th)_ = 1395.6, [M+2H^+^]^2+^_(exp)_ = 1395.0.

### 2.14 Synthesis of ZR‐A6–A11 lactam insulin (**8**)

The A‐chain *bis*‐SPy ZR‐A1–A21 lactam **6** (1.0 mg, 0.34 μmol) was dissolved in a buffer solution of 0.2 M Na_2_HPO_4_ and 6 M guanidine hydrochloride, pH 7. Recombinant B‐chain **7** (2.3 mg, 0.68 μmol) was added, and the solution was shaken for 1 h, followed by direct injection onto a HPLC column for purification (20%–80% buffer B over 60 min). About 0.3 mg of isomer ‘A’ and 0.4 mg of isomer ‘B’ was obtained, which indicates a combined yield of 31%. The yield of the parallel dimer (isomer ‘B’, **8**) was 18%. High‐resolution mass spectra of both isomers was conducted, and the monoisotopic molecular mass was determined. Isomer ‘A’: [M+4H^+^]^4+^_(th)_ = 1499.210, [M+4H^+^]^4+^_(exp)_ = 1499.210. Isomer ‘B’: [M+4H^+^]^4+^_(th)_ = 1499.210, [M+4H^+^]^4+^_(exp)_ = 1499.211.

### 2.15 Trypsin cleavage of the ZR motif

Protected (*S*‐Acm^A7^, *O*‐*t*Bu^A8^, *O*‐*t*Bu^A9^, *S*‐S*t*Bu^A20^) ZR‐A1‐A21 lactam (15.0 mg, 5.08 μmol) was dissolved in PBS buffer pH 7.4 (5 mg/mL), followed by the addition of trypsin (0.15 mg). The reaction was shaken for 1 h, followed by purification via preparative RP‐HPLC (20%–80% buffer B over 60 min). About 12.0 mg of purified product was obtained, indicating a yield of 87%. [M+2H^+^]^2+^_(th)_ = 1288.5, [M+2H^+^]^2+^_(exp)_ = 1288.0.

## 3 RESULTS AND DISCUSSION

### 3.1 Synthetic strategy

Our synthetic strategy (Scheme 1) involves ligation of the chemically synthesised protected A6–A11 fragment (**1**) with the A12–A21 fragment (**2**), followed by Fmoc deprotection to form the A6–A21 protected intermediate (**3**). A second ligation with the A1–A5 fragment (**4**) generates the full A‐chain protected intermediate **5**. Acid deprotection and concomitant *S*‐pyridylsulfenyl (*S*‐SPy) functionalisation of residues A7 and A20 then generates intermediate **6**. Finally, the A‐chain is combined with recombinant B‐chain (**7**) via thiolysis to form ZR‐A6–A11 lactam insulin (**8**). For this study, the A1–A5 and A12–A21 fragments were conveniently assembled via chemical synthesis, although it should be noted that both these peptide precursors can also be prepared via scalable recombinant techniques. The key challenge of this strategy is assembly of the A‐chain. This is because the A1–A5 and A12–A21 unprotected fragments—that contain multiple reactive functional groups—must be ligated chemoselectively to the chemically synthesised A6–A11 protected fragment. Of particular concern was the β‐thiol of cysteine at A20, which can react with active esters via its nucleophilic thiolate species to form thioesters and thus compete with the *N* ^α^‐amino group. To prevent this side reaction, the *S*‐*tert*‐butyl thiol (*S*‐S*t*Bu) group was introduced at A20 as thiol protection, which can be incorporated into unprotected recombinant peptides chemo‐selectively via asymmetric disulfide bond formation.^25^ Esterification via the N‐terminal serine A12 sidechain is another possible side reaction, although it is highly likely that the native backbone would subsequently be generated via an *O* → *N* acyl shift.^26^ An additional concern regarding A12–A21 is instability of the C‐terminal asparagine A21 residue, which could be deamidated during A‐chain assembly.^27^ To avoid this, asparagine was replaced with glycine, a substitution that is well‐tolerated and also present in insulin glargine.^28^ The A6–A11 macrocycle was isolated with base labile Fmoc *N* ^α^‐protection and acid‐labile sidechain protecting groups. This was done to prevent dimerisation via ester or thioester formation and to improve the solubility of the A‐chain intermediates in DMF, the solvent of choice for the fragment condensations.

Coupling A1A5 to the A6‐A21 fragment poses additional challenges due to the presence of the γ‐carboxylate of Glu^A4^, which prevents site‐selective activation of the C‐terminus. Therefore, the α‐carboxylate was replaced with a hydrazide moiety, which can be generated from a biosynthetic precursor via intein‐mediated hydrazinolysis.^29^ Hydrazides can be modified chemo‐selectively via reaction with sodium nitrite to form highly reactive acyl azides which are ideal for fragment condensations; however, glutaminyl α‐hydrazides have a propensity to form cyclic *bis*‐acyl hydrazides.^30^ To avoid this unproductive side reaction, activation of the A1–A5 C‐terminus was achieved via an acyl pyrazole intermediate (which can be generated via reaction of the hydrazide with acetoacetone) to form a stable selenoester.^31^ Unfortunately, introduction of a C‐terminal hydrazide prevents site‐selective *tert*‐butyloxycarbonyl (Boc) protection of the N‐terminus which is essential for chemo‐selective fragment condensation. Therefore, a pyroglutamyl‐arginyl (ZR) motif was installed at the N‐terminus, which can tolerate minor modification.^32^ Pyroglutamyl peptides can be readily generated through acid‐mediated cyclisation of N‐terminal glutamines^33^; introduction of the arginine maintains a positive charge proximal to the N‐terminus and also allows removal of the ZR dipeptide via enzymolysis using trypsin, if desired. After A‐chain assembly, combination with the B‐chain can then proceed via disulfide exchange, whereby formation of the correct parallel heterodimer is usually favoured.^15^

### 3.2 Synthesis of the A6–A11 protected fragment

**FIGURE 2:**
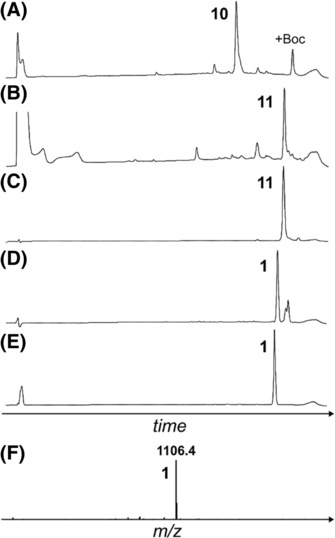
Analytical data of the protected ZR‐A6–A11 lactam fragment synthesis. (A) RP‐HPLC of crude **10** (linear material). (B) RP‐HPLC of the crude **11** (macrocyclic product). (C) RP‐HPLC of purified **11**. (D) RP‐HPLC of crude **1** (hydrolysed product). (E) RP‐HPLC of purified **10**. (F) ESI‐MS of purified **1** ([M+H^+^]^+^).

The macrocyclic A6–A11 protected peptide was synthesised on 2‐chlorotrityl resin as described in Scheme 2. The C‐terminal A11 aspartyl was anchored to the solid support via its β‐carboxylate sidechain to enable methyl protection of the C‐terminus which is required for site‐selective macrocyclisation. For the A7 cysteinyl sidechain, the diphenylmethyl (Dpm) group was used, which is more acid‐stable than the trityl (Trt) group.^34^ This stability is necessary for the subsequent Boc deprotection. After assembly on the solid support (**9**), the protected peptide was cleaved off the solid support with 3% trifluoracetic acid (TFA) in dichloromethane (DCM), which also selectively removed the Boc group on the A6 Dpr sidechain (**10**). The crude peptide was then cyclised via base‐catalysed activation to form the lactam between the A6 and A11 sidechains (**11**), followed by purification using reversed‐phase high‐performance liquid chromatography (RP‐HPLC). Selective removal of the C‐terminal methyl group in the presence of the *N* ^α^‐Fmoc group was then achieved using trimethyltin hydroxide,^35^ followed by further purification to obtain **1**. Please refer to Figure 2 for all relevant analytical RP‐HPLC data.

### 3.3 Assembly of ZR‐A6‐A11 lactam insulin

**FIGURE 3:**
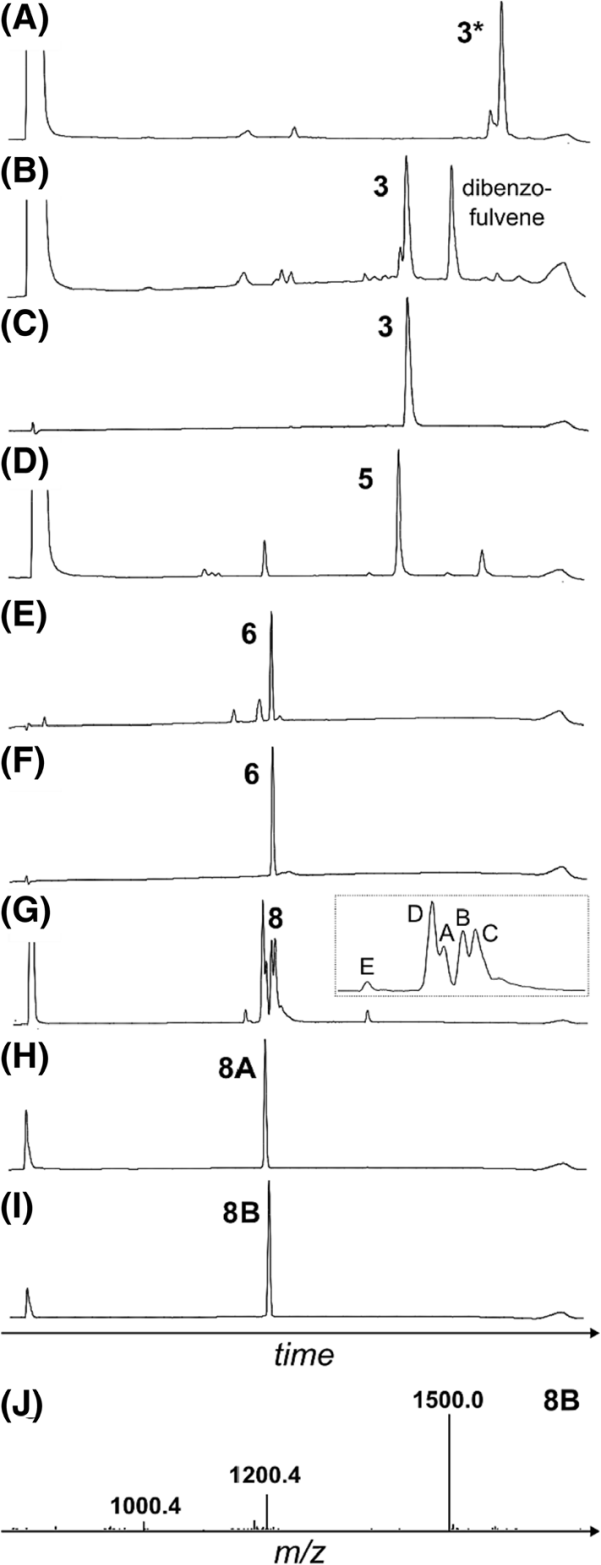
Analytical data of A6–A11 lactam insulin assembly. (A) RP‐HPLC of Fmoc‐protected 3*. (B) RP‐HPLC of crude **3** (Fmoc deprotected). (C) RP‐HPLC of purified **3**. (D) RP‐HPLC of protected **5**. (E) RP‐HPLC of crude **6** (*bis*‐pyridylsulfenyl A‐chain). (F) RP‐HPLC of purified **6**. (G) RP‐HPLC of crude **8** (two isomers). Zoomed inset: A = A6‐A11 lactam insulin isomer A (**8A**), B = A6 A11 lactam insulin isomer B (**8B**), C = reduced B‐chain, D = oxidised B‐chain, E = bicyclic A‐chain. **1**. (H) RP‐HPLC of purified **8A**. (I) RP‐HPLC of purified **8B**. J ESI‐MS of purified **8B** ([M+4H^+^]^4+^, [M+5H^+^]^5+^, [M+6H^+^]^6+^).

Detailed assembly of the A‐chain follows (refer to Scheme 1 and Figure 3). The C‐terminal carboxylate of the A6‐A11 protected fragment (**1**) was first pre‐activated in DMF using conventional base‐catalysed conditions, followed by the addition of the A12–A21 fragment (**2**). The reaction progressed slowly, which is likely due to the poor solubility of A12–A21 in DMF. Nevertheless, the coupling was complete in 18 h with only a minor side product formed, which was most likely the epimerised peptide given that it had the same molecular mass as the target peptide (Figure 3A). Piperidine was then added to the reaction mixture to effect Fmoc deprotection, followed by purification and lyophilisation to generate **3** (Figures 3B and 3C). The product was then redissolved in DMF, followed by the addition of the A1–A5 fragment (**4**), pre‐activated as the selenoester. This second reaction was complete after approximately 2 h, after which the protected A‐chain product (**5**) was purified. No evidence of epimerisation was observed for this second ligation although it cannot be ruled out.

Our original plan was to prepare *S*‐sulfonated A‐chain and combine it with the B‐chain (derived from recombinant human insulin) to form the desired heterodimer. However, when this was attempted, the major product observed was bicyclic A‐chain, ostensibly via an A7–A20 disulfide bond. This product also formed when *bis*‐thiol A‐chain was combined with *S*‐sulfonated B‐chain (data not shown). Therefore, the A‐chain was prepared as the *S*‐pyridylsulfenyl (*S*‐SPy) species to promote heterodimerisation. The SPy moiety is an excellent leaving group and is used routinely to form mixed disulfides, including for insulin analogues.^25^, ^36^ Formation of the A‐chain *S*‐SPy intermediate at A7 was achieved during the TFA cleavage of the remaining protecting groups with the addition of water and thioanisole as scavengers and 2,2′‐dipyridyl disulfide (DPDS). Analysis of the product determined that the *bis*‐*S*‐SPy product was formed; this was unexpected as the *S*‐S*t*Bu group at A20 was expected to be stable under these conditions. Nevertheless, the isolated product (**6**) was combined with excess B‐chain (**7**) to form both the parallel (**8**) and antiparallel heterodimeric products after 30 min, with the later eluting peak formed in a slightly higher proportion (Figure 3G). Both products were isolated and their in vitro biological activities were tested to determine the correct isomer (see below).

### 3.4 Analysis of ZR‐A6‐A11 lactam insulin

**FIGURE 4:**
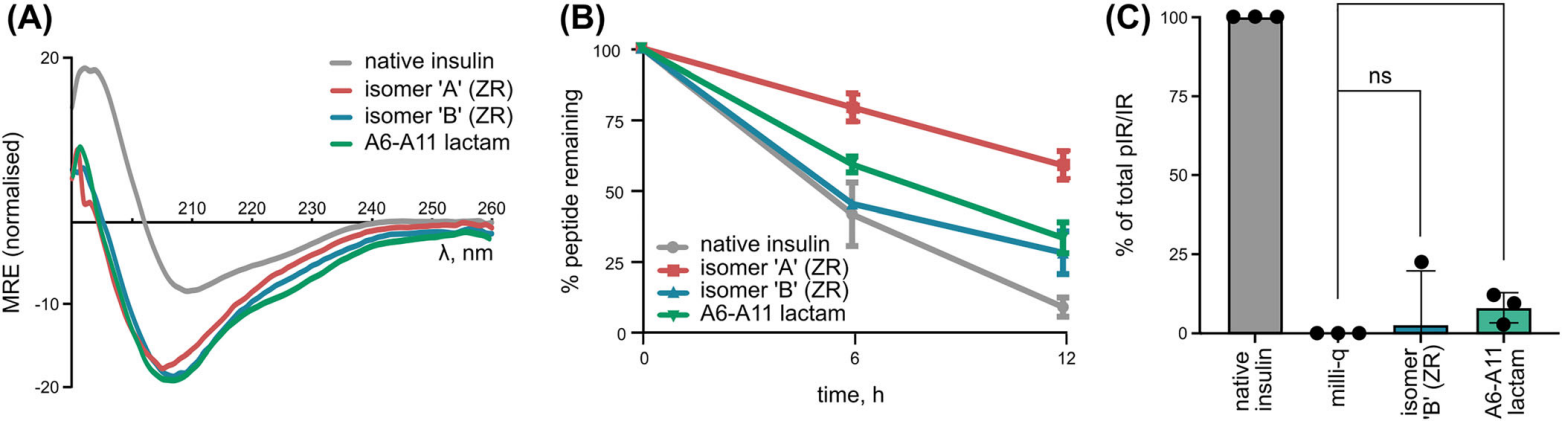
Analysis of the insulin lactam isomers and native insulin. (A) CD spectra. (B) Thermal stability assay (PBS at pH 7.4, 70°C). (C) IR phosphorylation.

Both insulin isomers were analysed via CD spectroscopy to probe the structural effect of incorporating the A6–A11 lactam bond (Figure 4A). Native insulin contains 3 α‐helical segments: two short segments on the A‐chain and one longer segment on the B‐chain. The spectra of both analogues are similar to one another and suggest that some α‐helical content is still present, with minima between 205 and 210 nm. However, the spectra noticeably differ to that of the spectrum of native insulin in the 190–200 nm range, which indicates a significant change in secondary and tertiary structure. The in vitro biological activity of the analogues was also determined in the Akt phosphorylation assay.^37^ Unfortunately, no significant activity was observed for both analogues based on Akt phosphorylation (Figure S1), although isomer ‘B’ may have low activity; the apparent poor activity is unsurprising given the change in protein conformation. Finally, the thermal stability of both isomers was benchmarked against native insulin. It was found that both lactam analogues are more stable than native insulin (Figure 4B), despite the significant structural perturbations imparted by the lactam bridge. Isomer ‘A’ was the most stable, with ~58% peptide remaining after 12 h, compared with ~27% for isomer ‘B’; only ~8% of native insulin remained over the same time period. This result further confirms the effectiveness of replacing the A6‐A11 disulfide bond with a more stable isostere for improved thermal stability.

**FIGURE 5:**
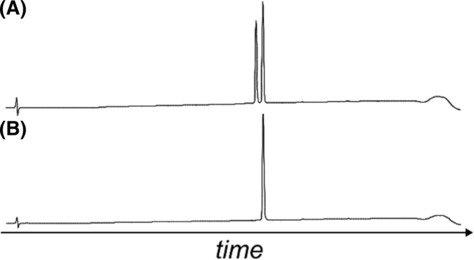
Chromatographic analysis of the ZR lactam insulin isomers co‐injected with the analogue formed through regioselective disulfide bond formation. (A) Isomer ‘A’. (B) Isomer ‘B’.

Unfortunately, these results are inconclusive regarding identification of the correct isomer. Therefore, the A6–A11 lactam analogue was resynthesised via regioselective disulfide bond formation using *S*‐acetamidomethyl (*S*‐Acm) protection for Cys A7 and B7 (see Section S2 in the supporting information), to ensure the correct disulfide connectivity. This strategy necessitates chemically synthesised B‐chain to site‐specifically incorporate the *S*‐Acm protecting group. After assembly, folding and isolation, the resynthesised ZR‐A6–A11 lactam insulin was co‐injected with isomers ‘A’ and ‘B’ (separately) onto an LCMS column, to determine the correct isomer. The chromatogram of isomer ‘A’ contained two peaks (Figure 5A), whereas the chromatogram of isomer ‘B’ contained one peak (Figure 5B). Therefore, isomer ‘B’ bears the correct disulfide connectivity.

A limitation of this study is the fact that the A6–A11 lactam insulin bears the ZR motif at the N‐terminus of the A‐chain, which could affect biological activity. Unfortunately, when attempting to cleave ZR from the full protein via trypsin, cleavage C‐terminal to Arg^B22^ was also observed as per Figure S2 (the expected cleavage C‐terminal to Lys^B29^ also occurred). Arg^B22^ typically forms a salt bridge with Glu^A17^ in native insulin and is thus resistant to trypsin's action^38^; however, this result suggests that the Arg side chain is exposed, due to the altered conformation of the lactam insulin. Therefore, the ZR motif was cleaved from the A‐chain, prior to the chain combination step (Figure S3). The resultant A6–A11 lactam insulin (Figure S4) was found to possess secondary structure similar to the other lactam analogues (Figure 4A) and comparable thermal stability (Figure 4B). There was a modest improvement in activity, with 3.8% Akt phosphorylation (Figure S1) and 8.0% IR phosphorylation (Figure 4C). Overall, though, this result suggests that the presence of the lactam bridge is still largely responsible for the significant reduction in biological activity.

## 4 DISCUSSION

The primary aim of this study was to demonstrate the feasibility of assembling an insulin analogue bearing a nonnative A6–A11 cystine isostere, using mostly biosynthetic precursors. This goal was achieved for the A‐chain assembly, given that the target compound for all synthetic steps was the major product. Although the total yield of ZR‐A6–A11 lactam insulin (**8**) is low—3.6% for both isomers and 2.0% for the correct parallel dimer (based on purified **1**)—the total yield of protected A‐chain fragment **5** was an impressive 24%; the total yield of *bis*‐SPy A‐chain **6** was 11%. There is of course significant scope for further refinement of the synthetic strategy (e.g., the chain combination step), which could lead to a scalable synthetic method. To improve the A‐chain synthesis, there are several options: (i) redesigning the protecting group strategy of the A6–A11 fragment to avoid both the challenging Boc deprotection and the methyl ester hydrolysis, (ii) incorporating additional protecting groups into the biosynthetic fragments to improve their solubility in DMF, (iii) minimising epimerisation during formation of the A6–A21 fragment (which is probably exacerbated by the lengthy reaction time), (iv) utilising a serine/threonine ligation^39^ (via the N‐terminal serine of the A12–A21 fragment) and (v) exploiting diselenide/selenoester ligations,^40^ which is chemoselective and can be conducted in aqueous media (this will address the solubility issue). It should be noted that handling losses will have significantly contributed to a reduction in yields, given that the reaction scales were only in the order of tens of milligrams.

Unfortunately, the chain combination step was challenging due to the apparent preference of the A‐chain to form a disulfide‐containing bicyclic structure. This result is at odds with the synthesis of the analogous A6–A11 diselenide analogue^15^ and likely due to the altered conformation the lactam bond imparts onto the insulin scaffold. To avoid the heterodimerisation step, we envisage that a proinsulin‐like precursor (with an enzyme cleavable C‐peptide) could be prepared in future, which would also assist in directing folding. Alternatively, a superior cystine isostere such as cystathionine^16^, ^17^— which does not significantly affect insulin's structure— would be more appropriate. A‐chain analogues such as these should be more amenable to the chain combination step.

Regarding the analogue itself, incorporation of the A6–A11 lactam bond has appeared to significantly alter the conformation of the A‐chain, as indicated by the CD spectroscopy data and the poor biological activity. This is likely due to a combination of factors relating to the amide bond structure, such as the shorter bond lengths and different bond angles within the modified crosslink, and a loss of conformational rigidity that the native disulfide crosslink imparts,^22^ such that the active three‐dimensional structure is adopted only transiently. Both lactam analogues are more thermally stable than native insulin and interestingly; the incorrect antiparallel ‘A’ isomer appears to be more thermally stable than the correct parallel ‘B' isomer. It is clear that better A6–A11 cystine mimetics, such as diselenide,^15^ cystathionine^16^, ^17^ and unsaturated *cis*‐dicarba^19^ bridges should be used in future work.

## 5 CONCLUSION

In summary, we have demonstrated proof of principle for the semisynthesis of insulin analogues bearing nonnative A6–A11 cystine isosteres. The key feature of the synthetic strategy involves the use of several biosynthetic precursors and a small, chemically synthesised A6–A11 macrocyclic fragment. The significant reduction in chemical steps suggests that insulin analogues such as these could be prepared on a large scale with further reaction optimisation, which promises to enhance their therapeutic potential. Although A6–A11 lactam insulin possesses poor biological activity, this synthetic strategy can be applied to other disulfide mimetics that have been shown to improve thermal stability or receptor selectivity without significantly affecting activity. We envisage that this new semisynthetic approach will also lead to the preparation of insulin analogues bearing A7–B7 and A20–B19 cystine isosteres. More broadly, this methodology can be applied to any disulfide‐rich protein and underpin the generation of a new class of hyper‐stable proteomimetics.

## Supporting information


**Figure S1.** Akt phosphorylation assay of isomers ‘A' and ‘B' (bearing ZR) and A6‐A11 lactam insulin.
**Scheme S1.** Synthesis of the heterodimer and subsequent iodine oxidation to produce A6‐A11 lactam insulin. **a** Aqueous 6 M guanidinium hydrochloride, 0.2 M Na_2_HPO_4_, pH 7, 25°C, 30 m. **b** Preparative RP‐HPLC. **c** I_2_, acetic acid, 50mM HCl_(aq)_, 25°C, 1 h. **d** Preparative RP‐HPLC.
**Figure S2.** Analytical data of the failed trypsin cleavage of isomer ‘B' (bearing ZR). **A** Analytical RP‐HPLC of the tryptic fragments. **B** ESI‐MS of peak A, which corresponds to B23‐B29. [M + H^+^]^+^_(th)_ = 860.0, [M + H^+^]^+^_(exp)_ = 859 .4. **C** ESI‐MS of peak B, which corresponds to A1‐A21/B1‐B22. [M + 3H^+^]^3+^_(th)_ = 1596.8, [M + 3H^+^]^3+^_(exp)_ = 1596.6.
**Figure S3.** Analytical data of the trypsin cleavage of protected (*S*‐Acm^A7^, *O*‐*t*Bu^A8^, *O*‐*t*Bu^A9^, *S*‐S*t*Bu^A20^) lactam A‐chain. **A** Analytical RP‐HPLC of the A‐chain bearing the ZR motif. **B** Analytical RP‐HPLC of the lactam A‐chain after trypsin cleavage. **C** ESI‐MS of the protected lactam A‐chain bearing the ZR motif. [M + 2H^+^]^2+^_(th)_ = 1422.2, [M + 2H^+^]^2+^_(exp)_ = 1421.8. **D** ESI‐MS of the lactam A‐chain after trypsin cleavage. [M + 2H^+^]^2+^_(th)_ = 1288.5, [M + 2H^+^]^2+^_(exp)_ = 1288.0.
**Figure S4.** Analytical data of A6‐A11 lactam insulin. A Pure RP‐HPLC data. B Pure ESI‐MS data. [M + 4H^+^]^4+^_(th)_ = 1433.4, [M + 4H^+^]^4+^_(exp)_ = 1433.2.
