# Supplementary material for: Semisynthesis of A6–A11 lactam insulin
Source: J Pept Sci. 2023 Sep 11;30(2):e3542. doi: 10.1002/psc.3542 (PMC10909544; doi:10.1002/psc.3542)
Supplement: Supplementary file 1 — Figure S1. Akt phosphorylation assay of isomers ‘A' and ‘B' (bearing ZR) and A6‐A11 lactam insulin. Scheme S1. Synthesis of the heterodimer and subsequent iodine oxidation to produce A6‐A11 lactam insulin. a Aqueous 6 M guanidinium hydrochloride, 0.2 M Na2HPO4, pH 7, 25°C, 30 m. b Preparative RP‐HPLC. c I2, acetic acid, 50mM HCl(aq), 25°C, 1 h. d Preparative RP‐HPLC. Figure S2. Analytical data of the failed trypsin cleavage of isomer ‘B' (bearing ZR). A Analytical RP‐HPLC of the tryptic fragments. B ESI‐MS of peak A, which corresponds to B23‐B29. [M + H+]+ (th) = 860.0, [M + H+]+ (exp) = 859 .4. C ESI‐MS of peak B, which corresponds to A1‐A21/B1‐B22. [M + 3H+]3+ (th) = 1596.8, [M + 3H+]3+ (exp) = 1596.6. Figure S3. Analytical data of the trypsin cleavage of protected (S‐AcmA7, O‐tBuA8, O‐tBuA9, S‐StBuA20) lactam A‐chain. A Analytical RP‐HPLC of the A‐chain bearing the ZR motif. B Analytical RP‐HPLC of the lactam A‐chain after trypsin cleavage. C ESI‐MS of the protected lactam A‐chain bearing the ZR motif. [M + 2H+]2+ (th) = 1422.2, [M + 2H+]2+ (exp) = 1421.8. D ESI‐MS of the lactam A‐chain after trypsin cleavage. [M + 2H+]2+ (th) = 1288.5, [M + 2H+]2+ (exp) = 1288.0. Figure S4. Analytical data of A6‐A11 lactam insulin. A Pure RP‐HPLC data. B Pure ESI‐MS data. [M + 4H+]4+ (th) = 1433.4, [M + 4H+]4+ (exp) = 1433.2. [file PSC-30-e3542-s001.docx]

**Supporting Information**

**Semisynthesis of A6-A11 lactam insulin**

Xu, R.,^a^ Jap, E.,^a^ Gubbins, B.,^b^ Hagemeyer, C. E.,^a*^ Karas, J. A.^b*^

*^a^Australian Centre for Blood Diseases, Monash University, Victoria, 3004, Australia*

*^b^School of Chemistry, The University of Melbourne, Victoria, 3010, Australia*

^*^Correspondence: [christoph.hagemeyer@monash.edu](mailto:christoph.hagemeyer@monash.edu), jkaras@unimelb.edu.au

**S1. Akt phosphorylation**


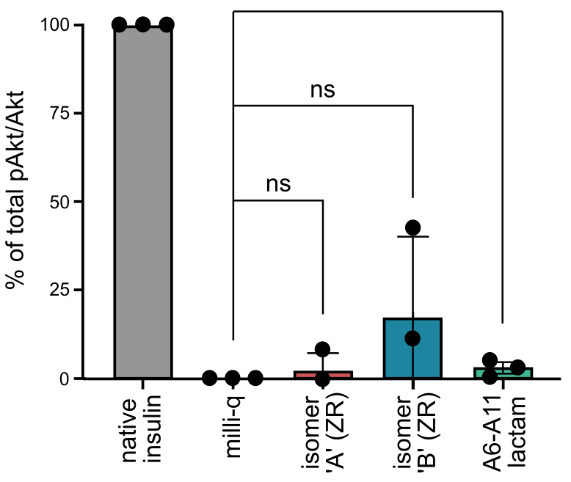


**Figure S1.** Akt phosphorylation assay of isomers ‘A’ and ‘B’ (bearing ZR) and A6-A11 lactam insulin.

**S2. Synthesis of A6‑A11 lactam insulin via regioselective disulfide bond formation**

**Scheme S1.** Synthesis of the heterodimer and subsequent iodine oxidation to produce A6-A11 lactam insulin. **a** Aqueous 6 M guanidinium hydrochloride, 0.2 M Na_2_HPO_4_, pH 7, 25°C, 30 m. **b** Preparative RP-HPLC. **c** I_2_, acetic acid, 50 mM HCl_(aq)_, 25°C, 1 h. **d** Preparative RP-HPLC.

**
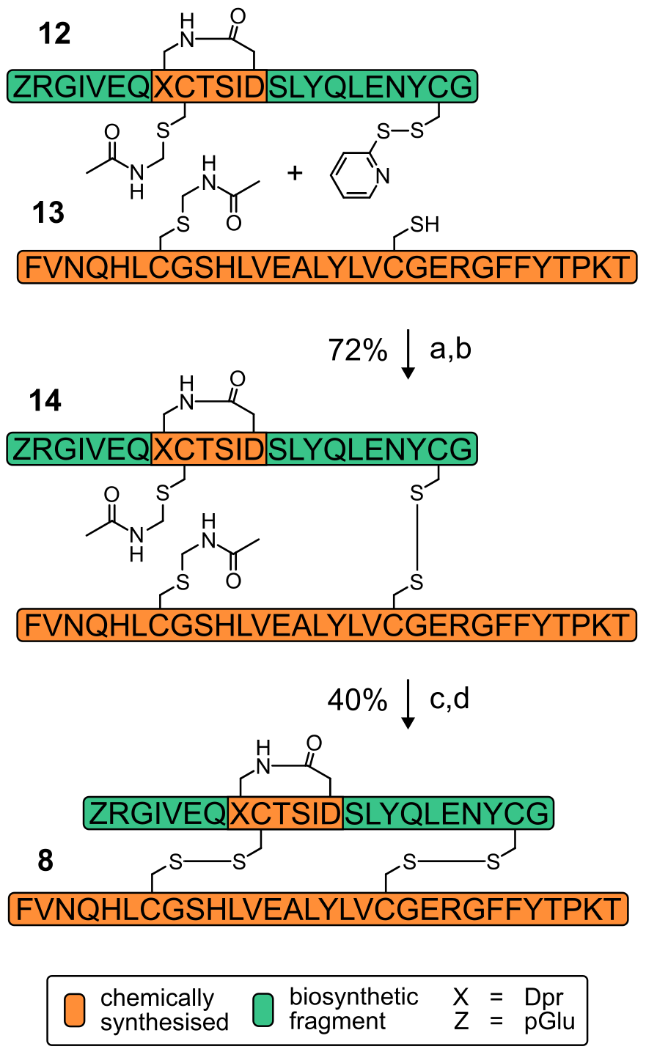
**

**S2.1 Synthesis of *S*-Acm^A7^, protected A6‑A11 lactam**

The *S*-Acm^A7^ linear peptide was assembled manually on a 0.3 mmol scale on 2-chlorotrityl resin using the same protocol as the analogous *S*-Dpm bearing precursor. After assembly, the resin was washed with 3% TFA in CH_2_Cl_2_ (10 x 10 mL) into a round bottomed flask, followed by stirring (18 h). The solvent was then reduced *in vacuo*, followed by dissolution in 50/50 acetonitrile/H_2_O and lyophilisation. The crude peptide mass was 347 mg, suggesting a yield of 100% based on the scale (likely to be inflated due to non-peptidic impurities). [M + H^+^]^+^_(th)_ = 1044.2, [M + H^+^]^+^_(exp)_ = 1044.4.

The crude *S*-Acm^A7^ peptide (347 mg, 0.3 mmol) was then dissolved in 95% CH_2_Cl_2_ containing 5% DMF (120 mL). DIEA (522 μL, 3.0 mmol) was then added, followed by the addition of HATU (342 mg, 0.9 mmol); the reaction mixture was stirred for 1 h. The solvent was then reduced *in vacuo* and the remaining DMF aspirated with nitrogen. The solid was then dissolved in 50/50 acetonitrile/H_2_O (25 mL), then purified (55-95% buffer B over 40 m). 67 mg of purified peptide was obtained, indicating a yield from the crude starting material of 22%. [M + H^+^]^+^_(th)_ = 1026.2, [M + H^+^]^+^_(exp)_ = 1025.4.

SnMe_3_OH (59.0 mg, 0.3265 mmol) was dissolved in dichloroethane (10 mL), followed by the addition of protected peptide methyl ester (67.0 mg, 0.0653 mmol). The reaction was stirred for 24 h at 55°C, followed by nitrogen gas aspiration. The remaining residue was then redissolved in 60% acetonitrile in H_2_O, then purified using a 45-95% buffer B gradient over 50 m. 23.0 mg of the free acid protected peptide was obtained, indicating a yield of 35%. [M + H^+^]^+^_(th)_ = 1012.2, [M + H^+^]^+^_(exp)_ = 1011.4.

**S2.2 Synthesis of *S*-Acm^A7^ protected A6‑A21 lactam**

The A6-A11 lactam peptide (23.0 mg, 22.7 μmol) was dissolved in dry DMF (2 mL), followed by the addition of COMU (9.7 mg, 22.7 μmol) and then NMM (10.0 μL, 90.8 μmol). The solution was shaken for 3 m, followed by the addition of the A12-A21 fragment (37.9 mg, 27.2 μmol). The reaction was shaken for 18 h and monitored by LCMS. Piperidine was then added, and the solution shaken for a further 10 m. The tube was then immersed in warm water and aspirated with nitrogen for 1 h to remove the DMF. The oily residue was then dissolved in acetonitrile/H_2_O (50/50) and purified via HPLC. Conditions: 40-90% buffer B over 50 m. 17.4 mg of pure A6-A21 protected lactam peptide was obtained, indicating a yield of 35%. [M + 2H^+^]^2+^_(th)_ = 1025.1, [M + 2H^+^]^2+^_(exp)_ = 1024.6.

**S2.3 Synthesis of *S*-Acm^A7^, *S*-SPy^A20^ ZR-A1‑A21 lactam (12)**

*S*-Acm^A7^ A6-A21 protected lactam peptide (17.4 mg, 8.05 μmol) was dissolved in dry DMF, followed by the addition of NMM (8.9 μL, 80.5 μmol). The ZR-A1-A5 selenoester peptide was then introduced (12.9 mg, 12.08 μmol) and the solution was shaken for 4 h, followed by nitrogen aspiration in a warm water bath for 1 h to remove the DMF. The crude oily residue was then treated with DPDS (26.6 mg, 120.75 μmol) in a solution of 1% thioanisole in TFA (2 mL) and shaken for 1 h. The TFA was reduced through nitrogen aspiration, followed by precipitation with diethyl ether and centrifugation. The white pellet was then solubilised in acetonitrile/H_2_O, filtered, and injected onto the RP-HPLC column for purification (20-80% buffer B over 60 m). 5.0 mg of the *S*-Acm^A7^, *S*-SPy^A20^, ZR-A1-A21 lactam peptide **12** was recovered, indicating a yield of 22%. [M + 2H^+^]^2+^_(th)_ = 1376.5, [M + 2H^+^]^2+^_(exp)_ = 1376.5.

**S2.4 Synthesis of *S*-Acm^B7^ B-chain (13)**

The peptide was assembled via microwave assisted SPPS on Wang resin preloaded with Fmoc-Thr(*t*Bu)-OH; Fmoc‑L-Cys(Acm)-OH was incorporated at position B7 (0.1 mmol scale). The peptide was cleaved from the resin with a cocktail of TIPS/H_2_O/TFA (2.5%/2.5%/95%) followed by filtration, nitrogen aspiration to reduce the volume, precipitation with diethyl ether, centrifugation, then decanting, to afford 203 mg of the crude peptide (yield = 50%). The crude material was purified using the following buffer system: buffer A = 10 mM NH_4_OAc at pH 8.5, buffer B = 10 mM NH_4_OAc in 80% acetonitrile_(aq)_ at pH 8.5. The gradient was 20-80% buffer B over 60 m. 24.7 mg of purified peptide **13** was recovered, indicating a yield of 12%. [M + 3H^+^]^3+^_(th)_ = 1168.0, [M + 3H^+^]^3+^_(exp)_ = 1167.8.

**S2.5 Synthesis of *bis*-*S*-Acm^A7,B7^ heterodimer (14)**

*S*-Acm^A7^, *S*-SPy^A20^ lactam A-chain **12** (5.0 mg, 1.75 μmol) was dissolved in a solution of 0.2 M Na_2_HPO_4_ and 6 M guanidine hydrochloride, pH 7 (2.7 mL) and acetonitrile (0.3 mL). *S*-Acm^B7^ B-chain **13** (8.5 mg, 2.1 μmol) was added, and the solution was shaken for 30 m, followed by direct injection onto a HPLC column for purification (20-80% buffer B over 60 m). 8.6 mg of the heterodimer (**14**) was obtained, indicating a yield of 72%. [M + 4H^+^]^4+^_(th)_ = 1536.2, [M + 4H^+^]^4+^_(exp)_ = 1536.2.

**S2.6 Synthesis of ZR-A6‑A11 lactam insulin (8)**

The *bis*-Acm heterodimer **14** (4.3 mg, 0.63 μmol) was dissolved in acetic acid (1 mL), followed by the addition of 50 mM HCl_(aq)_ (0.5 mL). Iodine (4.0 mg, 15.75 μmol) was dissolved in acetic acid (2 mL) and then added to the peptide solution. The reaction was mixture was shaken for 1 h and then quenched with ascorbic acid. The solution was then diluted with H_2_O up to 12 mL, then filtered and injected directly onto a HPLC column for purification (20-80% buffer B over 60 m). 1.7 mg of A6-A11 lactam insulin (**8**) was obtained in a yield of 40%. [M + 4H^+^]^4+^_(th)_ = 1500.2, [M + 4H^+^]^4+^_(exp)_ = 1500.0.

**S3. Trypsin cleavage of the ZR motif**

**
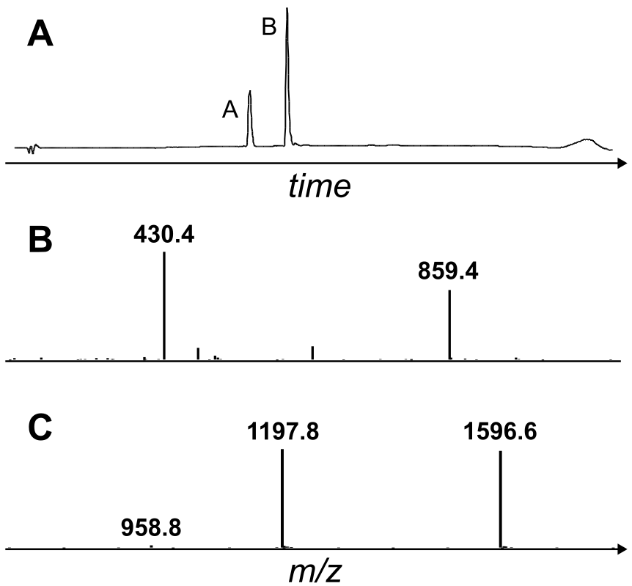
**

**Figure S2.** Analytical data of the failed trypsin cleavage of isomer ‘B’ (bearing ZR). **A** Analytical RP-HPLC of the tryptic fragments. **B** ESI-MS of peak A, which corresponds to B23-B29. [M+H^+^]^+^_(th)_ = 860.0, [M+H^+^]^+^_(exp)_ = 859 .4. **C** ESI-MS of peak B, which corresponds to A1-A21/B1-B22. [M+3H^+^]^3+^_(th)_ = 1596.8, [M+3H^+^]^3+^_(exp)_ = 1596.6.


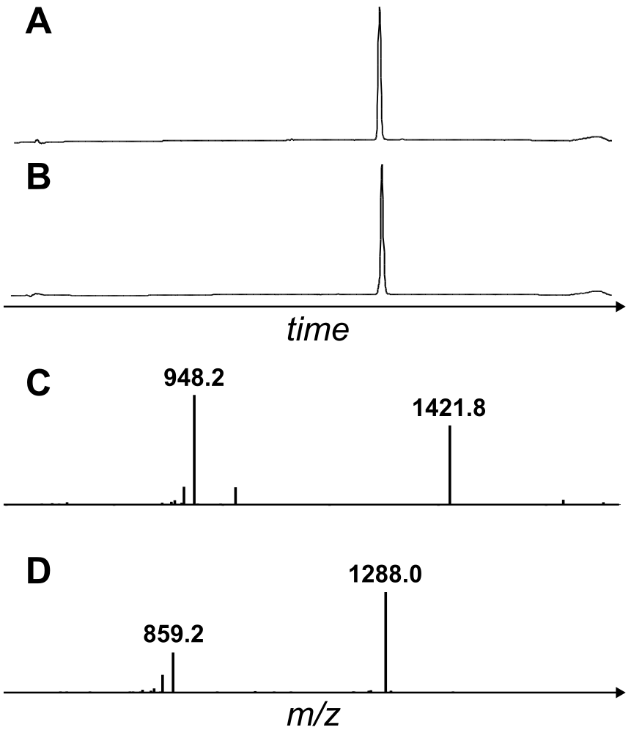


**Figure S3.** Analytical data of the trypsin cleavage of protected (*S*-Acm^A7^, *O*-*t*Bu^A8^, *O*-*t*Bu^A9^, *S*-S*t*Bu^A20^) lactam A-chain. **A** Analytical RP-HPLC of the A-chain bearing the ZR motif. **B** Analytical RP-HPLC of the lactam A-chain after trypsin cleavage. **C** ESI-MS of the protected lactam A-chain bearing the ZR motif. [M+2H^+^]^2+^_(th)_ = 1422.2, [M+2H^+^]^2+^_(exp)_ = 1421.8. **D** ESI-MS of the lactam A-chain after trypsin cleavage. [M+2H^+^]^2+^_(th)_ = 1288.5, [M+2H^+^]^2+^_(exp)_ = 1288.0.

**S4. A6-A11 lactam insulin**

**
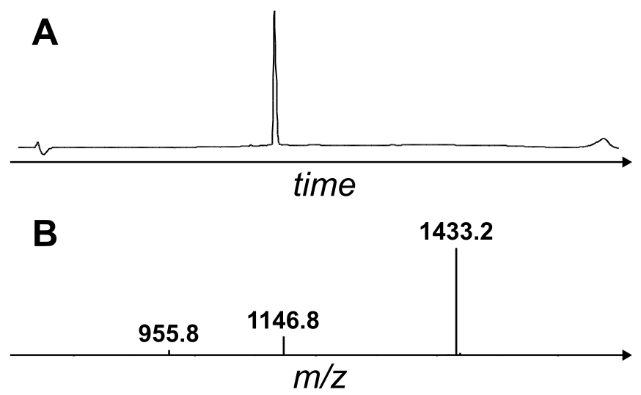
**

**Figure S4.** Analytical data of A6-A11 lactam insulin. **A** Pure RP-HPLC data. **B** Pure ESI-MS data. [M + 4H^+^]^4+^_(th)_ = 1433.4, [M + 4H^+^]^4+^_(exp)_ = 1433.2.
